# Ischemic Left Ventricular Perforation Covered by a Thrombus in a Patient Presenting with Cerebral Ischemia: Importance of Time-Sensitive Performance and Adequate Interpretation of Bedside Transthoracic Echography

**DOI:** 10.1155/2016/7565042

**Published:** 2016-02-04

**Authors:** A. J. Fischer, P. Lebiedz, M. Wiaderek, M. Lichtenberg, D. Böse, S. Martens, F. Breuckmann

**Affiliations:** ^1^Department of Cardiovascular Medicine, Division of Electrophysiology, University of Münster, 48149 Münster, Germany; ^2^Department of Cardiovascular Medicine, University of Münster, 48149 Münster, Germany; ^3^Department of Neurology, Arnsberg Medical Center, 59759 Arnsberg, Germany; ^4^Department of Angiology, Arnsberg Medical Center, 59759 Arnsberg, Germany; ^5^Department of Cardiology, Arnsberg Medical Center, 59759 Arnsberg, Germany; ^6^Department of Cardiothoracic Surgery, Division of Cardiac Surgery, University of Münster, 48149 Münster, Germany

## Abstract

If myocardial infarction remains silent, only clinical signs of complications may unveil its presence. Life-threatening complications include myocardial rupture, thrombus formation, or arterial embolization. In the presented case, a 76-year-old patient was admitted with left-sided hemiparesis. In duplex sonography, a critical stenosis of the right internal carotid artery was identified and initially but retrospectively incorrectly judged as the potential cause for ischemia. During operative thromboendarterectomy, arterial embolism of the right leg occurred coincidentally, more likely pointing towards a cardioembolic origin. Percutaneous interventions remained unsuccessful and local fibrinolysis was applied. Delayed bedside echocardiography by an experienced cardiologist demonstrated a discontinuity of the normal myocardial texture of the left ventricular apex together with an echodense, partly floating structure merely attached by a thin bridge not completely sealing the myocardial defect, accompanied by pericardial effusion. The patient was immediately transferred to emergency cardiac surgery with extirpation of the thrombus, aortocoronary bypass graft placement, and aneurysmectomy. This didactic case reveals decisive structural shortcomings in patient's admission and triage processes and underlines, if performed timely and correctly, the value of transthoracic echocardiography as a noninvasive and cost-effective tool allowing immediate decision-making, which, in this case, led to the correct but almost fatally delayed diagnosis.

## 1. Introduction

Myocardial infarction is one of the major causes of death in the industrialized world. It is characterized as an ischemic necrosis of cardiac cells in a clinical setting consistent with acute myocardial ischemia [1]. Patients with myocardial infarction may present with different symptoms ranging from epigastric pain to typical left thoracic chest pain. As little as 40% of patients present with typical clinical signs of myocardial infarction [2]. Therefore, diagnosis is often delayed as to the time patients present with clinical signs of complications of myocardial infarction only. There are several severe complications worsening the overall prognosis. Myocardial rupture that can appear in the acute setting with laceration of the myocardium represents a main life-threatening complication. Fibrinolytic agents should not be administered as it has been shown that cardiac rupture may be accelerated by thrombolytic therapy, especially in case a thrombus seals the perforated myocardium by adherence to the pericardium [3]. Another serious complication is left ventricular thrombus formation. After myocardial infarction, wall motion abnormalities may lead to stagnated blood flow and thus ventricular thrombus formation. After confirmation of a left ventricular thrombus, systemic anticoagulation is the treatment of choice in addition to antiplatelet therapy, reducing the incidence of embolism [1, 4, 5].

If performed and adequately interpreted, transthoracic echocardiography is an adequate diagnostic tool to visualize ventricular thrombi [4, 6]. However, especially if myocardial infarction remains silent within the acute setting, only ventricular thrombus embolization unveils its presence.

## 2. Case Presentation

**Figure 1:**
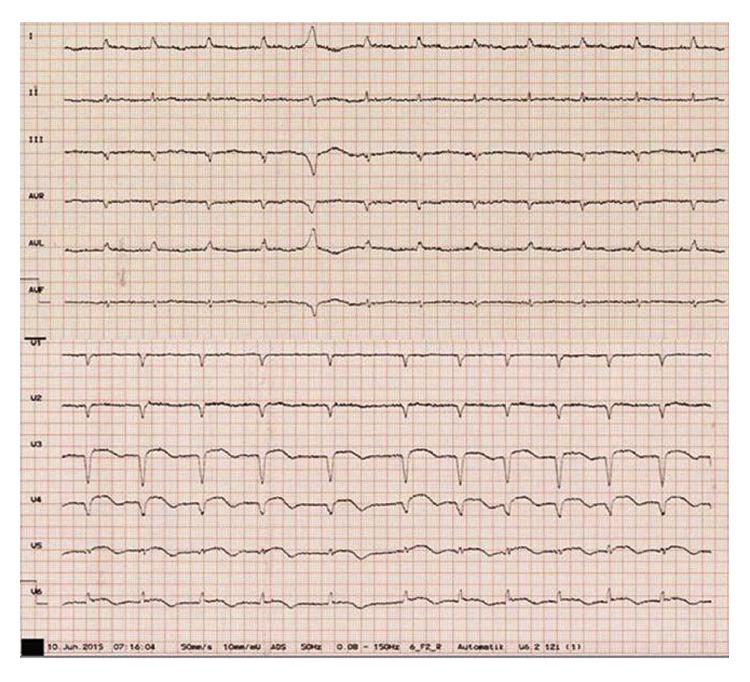
Initial twelve-lead ECG demonstrating atrial fibrillation and ST-segment elevation of the anterior leads.

**Figure 2:**
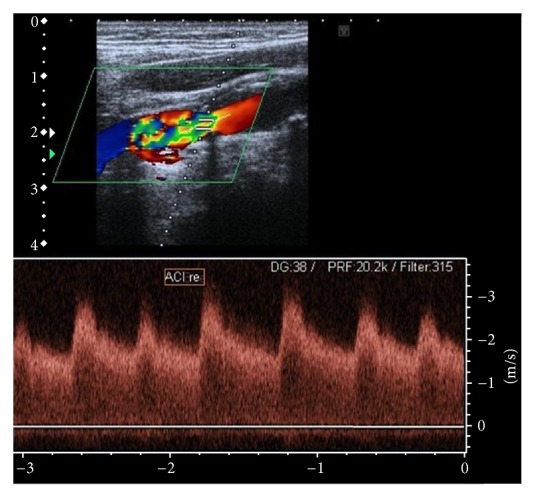
Doppler ultrasound assessment of the right internal cerebral artery showing a critical stenosis with a systolic maximal flow velocity of >3 m/s.

**Figure 3:**
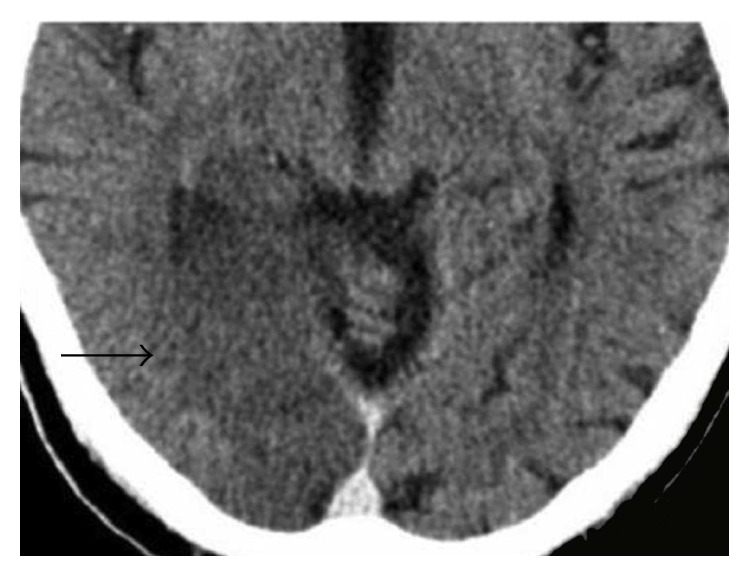
Contrast-enhanced cranial computed tomographic scan showing an insult of the right posterior region with hypodensity of the parafalcine parenchyma as well as loss of grey/white matter differentiation (black arrow).

**Figure 4:**
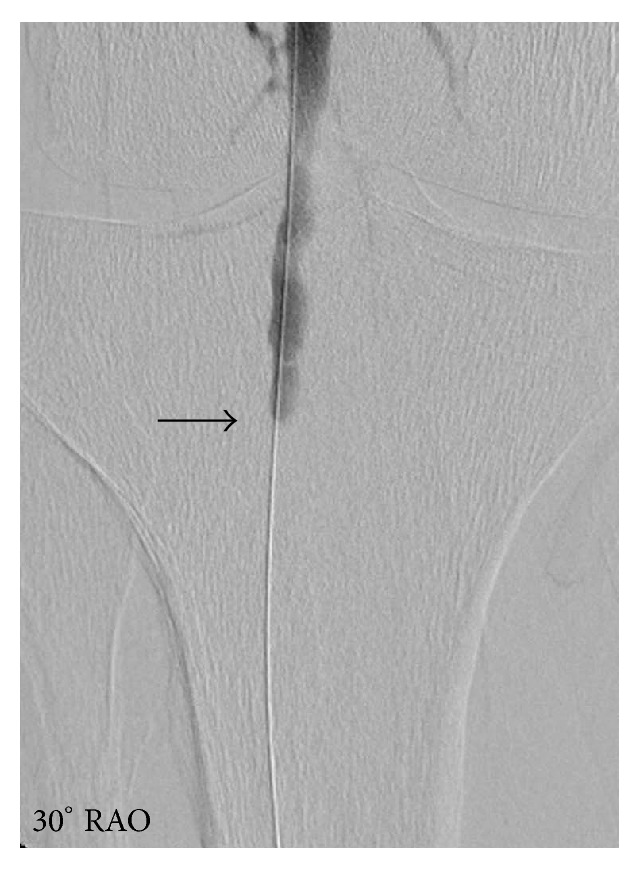
Percutaneous transluminal angiography demonstrating the occlusion of the right popliteal artery (black arrow).

**Figure 5:**
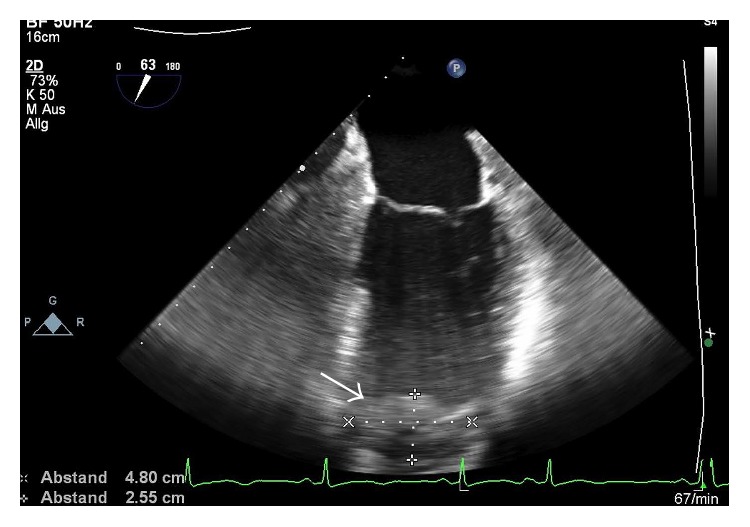
Transesophageal echocardiographic assessment of the left ventricular thrombus in the two-chamber view. The thrombus is marked with a white arrow.

**Figure 6:**
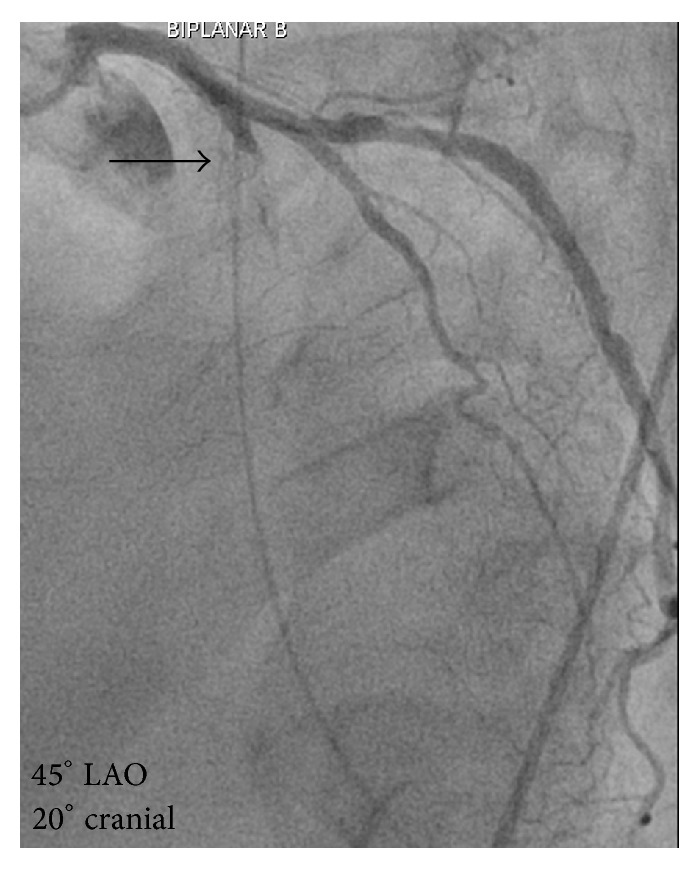
Coronary angiography revealing a complete occlusion of the left anterior descending coronary artery marked with the black arrow.

**Figure 7:**
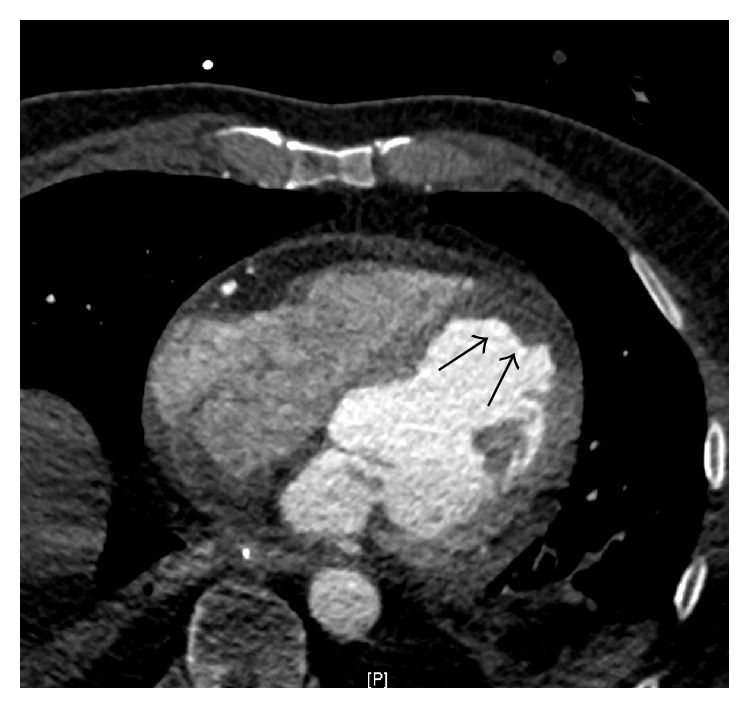
Contrast enhanced ECG-gated chest computed tomographic scan showing loosening of the myocardial anteroapical wall as well as thrombus formation (black arrow).

**Figure 8:**
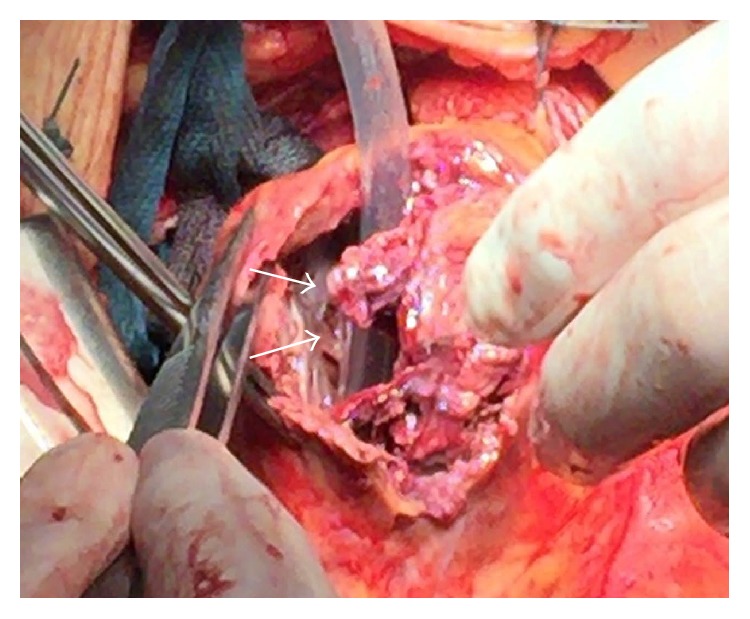
Intraoperative situs showing an ischemic myocardial perforation of the left ventricle.

A 76-year-old patient presented with left-sided hemiparesis to our neurologic department. Clinical assessment revealed a NIH Stroke Scale of eight. Electrocardiogram at admission showed atrial fibrillation concomitant with ST elevation in the anterior leads V3–V6 (Figure 1). The initial native cranial computed tomography (CT) did not show significant changes. As the patient reported that symptoms persisted since the day before presentation, potential lysis therapy was not an option and additional contrast-enhanced scans were omitted. Instead, because of a positive history of vascular disease, early carotid as well as transcranial duplex was performed. A stenosis of the right internal carotid artery of about 80% was diagnosed and considered as origin for the acute cerebral ischemia (Figure 2). As to progressive left-sided hemiparesis, it was decided to perform operative thromboendarterectomy of the right carotid artery immediately. During surgery, coincident embolization of the right leg occurred. Bedside transthoracic echocardiography by a nonprofessional observer disclosed signs of chronic myocardial infarction and a suspicious, partly free-floating echodense structure attached to the thinned anterior wall. Retrospectively, cardioembolic stroke due to atrial fibrillation or left ventricular thrombus was now assumed. Repeated cranial CT confirmed ischemia in the posterior flow area and pointed towards simultaneous ischemic brain stem stroke (Figure 3). Duplex sonography of the right leg revealed an occlusion of the femoral artery. Therefore, the patient was subsequently transferred to percutaneous transluminal angioplasty (Figure 4). Up to this point, only unfractionated heparin was administered for anticoagulation. As during the interventional procedure perfusion of the leg remained impaired, local fibrinolysis was applied as bail-out. Due to persisting insufficient perfusion, operative popliteopedal bypass surgery had to be performed finally. In the intensive care unit, an experienced cardiologist repeated postinterventional transthoracic echocardiography. Imaging showed a severely impaired left ventricular function accompanied by a discontinuity of the normal myocardial texture of the apex together with an echodense, partly floating structure merely attached by a thin bridge, not completely sealing the assumed myocardial defect anymore. Simultaneously, a progression of the pericardial effusion had occurred. Transesophageal echocardiography confirmed transthoracic suspicion (Figure 5). Imminent myocardial rupture was feared and urgent cardiac surgery was planned, accepting an elevated risk of secondary cerebral bleeding. Preoperative coronary catheterization showed a complete occlusion of the proximal left anterior descending artery (Figure 6). Cardiac CT imaging showed a thrombus with exophytic components into the left ventricle adherent to a postischemic anterior aneurysm with extremely thinned myocardium (Figure 7). Surgery validated a perforated left ventricle partly covered by a thrombus. An extirpation of the thrombus and coronary bypass graft were performed (Figure 8). The apical aneurysm was resected. Endomyocardial biopsies revealed thrombotic material along with fibrosis.

In the following days, the perfusion of the right leg remained critically impaired despite reoperation. Ultimately, an amputation of the right lower limb got necessary. A hemiparesis persisted. Transthoracic echocardiographic follow-up before discharge revealed that the patients' left ventricular ejection fraction had improved without any more thrombus formation.

## 3. Discussion

Often, clinical signs of myocardial infarction are unspecific, particularly in females, elderly, diabetics, and patients with dementia or suffering from chronic renal disease [1, 7]. As a consequence, patients may present in a subacute setting with clinical signs of complications as in the presented case.

Particularly, myocardial infarction of the anterior wall can lead to thrombus formation because of apical aneurysm and, thus, stagnant blood flow in the apex. In a multicenter trial on patients after acute myocardial infarction who were considered in low to medium risk for left ventricular thrombi, in 5.1%, a thrombus could be diagnosed within predischarge echocardiogram. In anterior myocardial infarction, there was even an incidence of 11.5% [8]. If the thrombus is free-floating within the left ventricle, there is a considerable risk of arterial embolism. In fact, the risk for cardioembolic events is fivefold higher in patients after myocardial infarction with detected thrombus formation as compared to patients without echocardiographic signs of thrombus formation [5]. Even though these cardioembolic events are well known, early bedside transthoracic echocardiogram is, as in our case, not yet implemented in clinical practice. Our case, which can be considered as a teaching case, demonstrates the value of transthoracic echocardiography as a noninvasive and cost-effective tool for immediate decision-making, at least within experts' hands. At initial presentation of the patient, an ECG was performed where atrial fibrillation as a potential cause for thromboembolism and ST elevation of the anterior leads as a sign for myocardial ischemia or myocardial aneurysm were detected. Because of structural failures in a common setting without central interdisciplinary emergency department but in case of leading neurological symptoms direct admission to the stroke unit, neurological work-up was overweighed and the ECG was not given sufficient attention. Even though it has been shown that particularly in patients with intracerebral hemorrhage but also with ischemic stroke ECG changes such as ST-segment elevation can be present, however, an echocardiography directly at admission or prior to surgery performed by an experienced physician may have led to much earlier diagnosis of imminent myocardial rupture, preventing unnecessary surgery of the internal carotid artery and further systemic embolization [9]. Most dramatically, orientating echocardiography by a noncardiologist even was performed within the clinical work-up; however, the information gathered was misinterpreted or not correctly weighted, particularly when local fibrinolysis was applied in the later course. Even though anticoagulation is the adequate treatment for prevention of thromboembolic events when left ventricular thrombus has been detected, thrombolytic therapy should not be administered [1, 4]. In the presented case, inadequate application of fibrinolytic agents resulted in an almost fatal rupture of the myocardium.

Nonetheless, it would be inappropriate to blame rescue fibrinolytic therapy initiating this sort of medical nightmare. By contrast, the presented case initiated internal discussions and review of procedural shortcomings in the aforementioned common patient population, thereby helping us to improve processes in our hospital. We learned that echocardiography has to be performed early in the clinical course in patients with seemingly unrelated clinical symptoms and integrated early ECG and echocardiography assessment within our stroke unit protocols. Even in acute and urgent cases, where a delay of operative treatment may potentially lead to more neurological damage, at least a short cardiologic consultation should be prompted whenever there are hints for a cardioembolic origin. Otherwise, potentially life-threatening illnesses may be missed.
